# State Medical Society

**Published:** 1874-02

**Authors:** 


STATE MEDICAL SOCIETY.—The Annual Meeting of the State Medical Society was largely attended and was full of interest. The Society voted to try the experiment of publishing its own transactions, in which determination we hope it will be encouraged by the county societies. The following officers were elected for the ensuing year.

*President*, Dr. Geo. J. Fisher, Sing Sing.
*Vice President*, Dr. Harvey Jewett, Canandaigua.
*Secretary*, Dr Wm. H. Bailey, Albany.
*Treasurer*, Dr. Charles H. Porter, Albany.

